# What Temperature of Coffee Exceeds the Pain Threshold? Pilot Study of a Sensory Analysis Method as Basis for Cancer Risk Assessment

**DOI:** 10.3390/foods7060083

**Published:** 2018-06-01

**Authors:** Julia Dirler, Gertrud Winkler, Dirk W. Lachenmeier

**Affiliations:** 1 University of Applied Sciences Albstadt-Sigmaringen, Department Life Sciences, Anton-Günther-Str. 51, 72488 Sigmaringen, Germany; julia.dirler@web.de (J.D.) winkler@hs-albsig.de (G.W.); 2 Chemisches und Veterinäruntersuchungsamt (CVUA) Karlsruhe, Weissenburger Strasse 3, 76187 Karlsruhe, Germany

**Keywords:** coffee, hot beverages, temperature, esophageal cancer, thermosensing, sensory thresholds, methodological study

## Abstract

The International Agency for Research on Cancer (IARC) evaluates “very hot (>65 °C) beverages” as probably carcinogenic to humans. However, there is a lack of research regarding what temperatures consumers actually perceive as “very hot” or as “too hot”. A method for sensory analysis of such threshold temperatures was developed. The participants were asked to mix a very hot coffee step by step into a cooler coffee. Because of that, the coffee to be tasted was incrementally increased in temperature during the test. The participants took a sip at every addition, until they perceive the beverage as too hot for consumption. The protocol was evaluated in the form of a pilot study using 87 participants. Interestingly, the average pain threshold of the test group (67 °C) and the preferred drinking temperature (63 °C) iterated around the IARC threshold for carcinogenicity. The developed methodology was found as fit for the purpose and may be applied in larger studies.

## 1. Introduction

Since 2016, the cancer risk in connection to hot beverage consumption has received increased scrutiny from science and consumers alike. The reason for this has been the classification of “very hot beverage consumption” by the International Agency for Research on Cancer (IARC) into group 2A as “probably carcinogenic to humans” [1,2]. Specifically, the risk of developing oesophageal carcinoma increases with the consumption of very hot beverages as shown by a number of epidemiological studies [3,4,5,6,7,8]. Beverages above 65 °C are considered “very hot” [1,2].

There are only a few studies available that researched the perception of temperature when consuming hot drinks. In general, the thermoreceptors are responsible for the sensation of heat and cold. These receptors are located in the skin and mucous membranes. When an action potential occurs, these receptors relay the stimulus to the nervous system, triggering a sensation [9,10]. The thermoreceptors are located 0.1 to 0.6 mm below the skin surface, in the dermis. These receptors are located not only on the surface of the skin, but also inside the body, e.g., on the internal organs and their mucous membranes [11]. The thermoreceptors can be divided into cold and warm ones. These react during cooling or warming with an impulse increase, thus leading to an action potential. More specifically, transient receptor potential (TRP) channels sense hot and cold. TRP channels respond to stimuli from temperature, pressure, inflammatory agents, and receptor activation. TRP cation channel subfamily V member 1 (TRPV1) receptors open at temperatures greater than 43 °C. TRP cation channel subfamily M member 8 (TRPM8) channels sense cooling, and open at temperatures <26 °C [12,13]. TRPV1 and TRPM8 channels have unusually large Q_10_ values (>15 for TRPV1) [12,14,15].

Important factors in the sensation of temperature are the absolute temperature, the steepness of the change in the temperature which affects the skin during a certain time and the size of the irritated body surface. In addition, the thermal conductivity of the object or the fluid plays a role [11]. Above temperatures of about 44–45 °C, the human begins to develop a painful heat sensation. Pain stimuli are absorbed by pain receptors. These receptors are located in the epithelia of the skin and mucous membranes. These receptors do not approach a specific organ but run in the intercellular clefts of the epithelium. The pain receptors react differently to different stimuli, e.g., on heat, pressure and strain [11]. Only after a series of action potentials and the exceedance of the threshold value, a pain stimulus is triggered [11]. At higher temperatures (surface temperatures of >70 °C for less than one second), trans-epidermal necrosis may occur [16].

The increase in the temperature at the tongue surface results from the contact temperature between the liquid and the tongue. The contact temperature is the point at which two bodies touch each other at different temperatures. This temperature can be estimated by the simple Formula (1) [17]:

(1) $$T_{K}=T_{2}+\frac{b_{1}}{b_{1}+b_{2}}*\left(T_{1}-T_{2}\right)$$

where *T_K_* = Contact temperature, *b*_1/2_ = Thermal effusivity, *T*_1/2_ = Temperature of body ½. 

The skin temperature is about 37.4 °C with a thermal effusivity of about 1.3 kWs^0.5^m^−2^K^−1^. The thermal effusivity of water, on the other hand, is 1.6 kWs^0.5^m^−2^K^−1^ [17]. If the coffee has a temperature of 70 °C, thus the contact temperature estimation will be about 55 °C. On the other hand, the formula results in a temperature of 57 °C, which would cause the tongue to be heated at around the pain threshold temperature of 48 °C [18]. This is corroborated by the optimal drinking temperature of 57.8 °C postulated by Brown and Diller [19] based on data modelling for simulating burns from various in vivo studies. These theoretical estimations well confirm the measurements of proband’s tongue surfaces of Lee et al. [18]. Recommended maximum temperatures for water to avoid burning were 65 °C for contact periods up to 1 s duration and 60 °C for contact periods of 3–4 s [20].

Only a few experimental approaches are available for the determination of drinking temperatures by means of sensory tests. Graham et al. [21] adapted the method of Pearson and McCloy [22] to estimate the preferred drinking temperature of hot drinks. Hot water, with an initial temperature of 80 to 85 °C, was filled in a porcelain cup. Each time the water cooled down 2 °C, participants were asked to sample the water and give their assessment of the current temperature [21]. Another study also determined which temperatures of the hot drinks are perceived as preferable by consumers. For this purpose, a mixing method of coffee with different temperatures has been developed. The participants were asked to mix their coffee to that temperature they usually would consume the beverage. At first, very hot coffee has been in the cup to be tasted, which was gradually mixed with colder coffee. Having reached the optimum temperature of the coffee, the temperature was measured and documented [23]. Borchgrevink et al. [24] used a similar design but with randomized sequence of temperatures between 57 °C and 91 °C aiming to minimize the potential of an order or undesired treatment effect. In even another design, Pipatsattayanuwong et al. [25] presented six different temperatures ranging from 39 to 82 °C but the two central temperatures (61 °C and 72 °C) were to be tasted first to avoid initial tasting of extremes.

The aim of this study was to develop a method to elucidate which temperatures of hot beverages are perceived as too hot. That is the temperature at which consumers can no longer drink the coffee without feeling pain. Since published reports were open to interpretation, we developed a new method based on the study of Lee and O’Mahony [23] but with an inverse experimental design because we judged it as being inappropriate to start from the pain stimulus directly in the very first tasting. The randomized design of Borchgrevink et al. [24] would also not completely avoid this effect as some participants may still begin with potentially scalding beverages with the potential to make them unable to adequately assess the following lower temperature beverages. Therefore, in our design the temperatures of coffee are gradually increased until the pain stimulus level is reached. For a pilot study of the method, coffee (standard caffeine-containing type) is used as model because it is the most commonly consumed hot beverage in Germany and no data at all regarding this question is available for the German population. 

## 2. Materials and Methods 

The mixing method according to Lee and O’Mahony [23] is used with some modifications. In contrast to the reduction of temperature in the original protocol, the coffee temperature is gradually increased by adding very hot coffee until the participants perceive the drink as being too hot. In each step, the participants re-test the hot drink after a temperature increase of 2–3 °C. 

**Figure 1:**
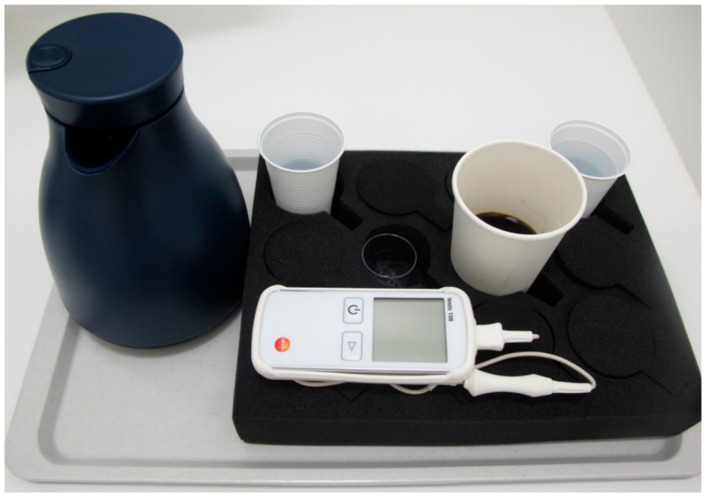
Experimental set-up provided to each participant. The isolation cup contains 150 mL of coffee at 60 °C and the thermos flask coffee at 93 °C. The small 40 mL beaker cup is used to gradually add the hot coffee. Two further cups with cold water for spilling if required and an empty cup for spitting are provided. The digital thermometer was used to measure the temperature in each tasting step.

The experimental setup is shown in Figure 1. Each participant receives a cup of cold water, a cup as spittoon, a thermometer (Testo 108, Testo, Lenzkirch, Germany), a beaker, a thermos flask with very hot coffee (Instant coffee, Brand “Gut und Günstig”, Edeka, Hamburg, Germany) and an isolation cup (Styrofoam cup, 400 mL Thermo Cup EPS Neutral, white, Gastro-Sun.de, Blankenhain, Germany) with the “colder” coffee.

**Figure A1:**
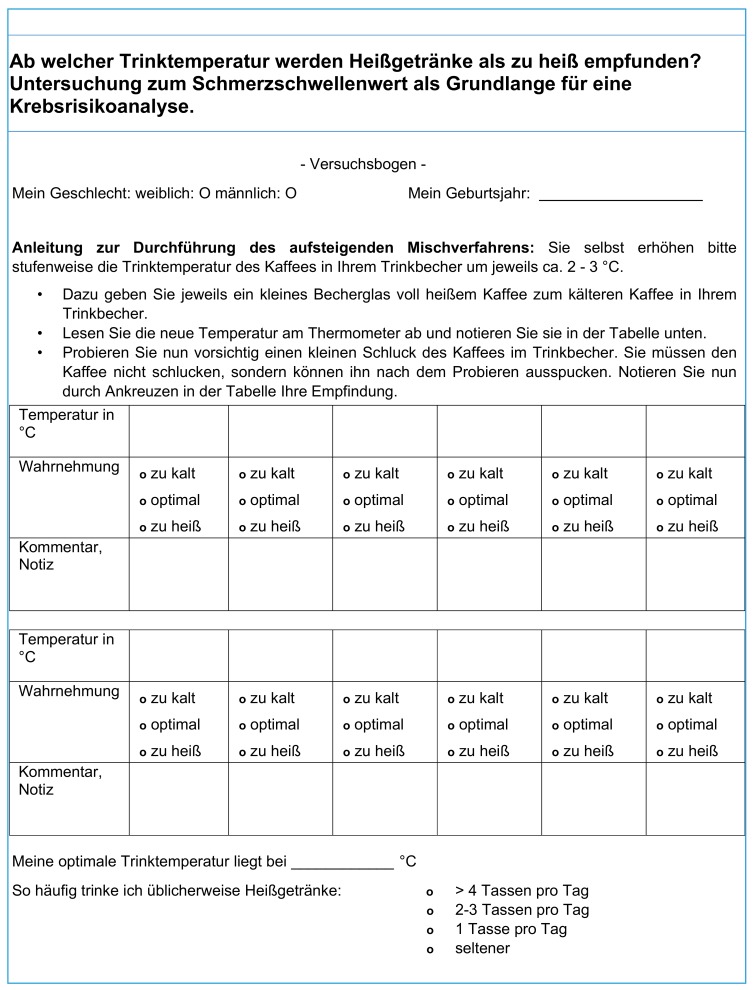
Experimental sheet for each participant of the trial (original in German).

**Figure A2:**
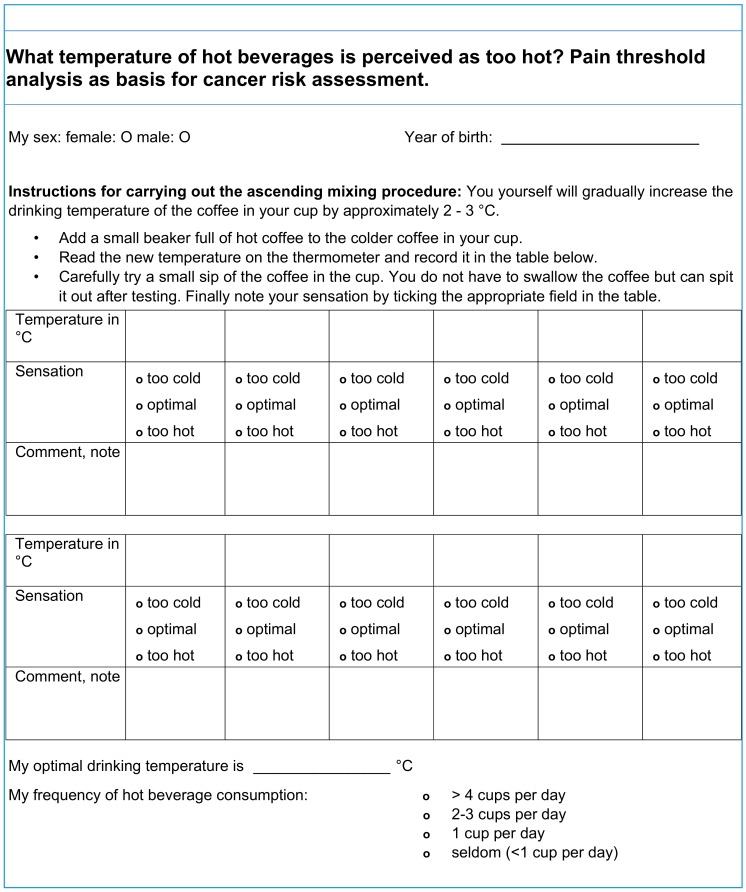
Experimental sheet for each participant of the trial (English translation).

Before the start of the study, the participants are trained through a short briefing so that all of them have understood the test procedure and any questions that may arise can be clarified before the start of the test. The test sheet (Figure A1 and Figure A2, Appendix A), which is given to the participant to be filled in during the test run, also contains the test instructions. Then it contains a table, in which the temperatures of the tasted coffees are entered. For each tasted temperature of the hot drink, the participants give a judgement about the taste sensation using a three-category scale (with the word descriptors: too cold, optimal, or too hot) and may make a brief remark in their own words. Finally, the participants indicate how often they usually ingest hot drinks.

The coffee is prepared each time with the same amount of tap water (200 mL) and soluble coffee powder (4.5 g). To ensure a similar starting temperature for all participants, a pot of coffee is prepared 15 min before they arrive. This coffee is cooled down to the desired temperature using a thermostatted water bath with temperature control. The desired starting temperature for all participants is 60 °C.

The very hot coffee, however, is poured only shortly before the start of the experiment to ensure its high temperature. In order to be able to hinder the rapid cooling of the coffee, tests are carried out with a cup holder made of foam (Florence foam holder for 12 glasses/cups, 330 × 245 × 60 mm, Haba BV, Maasland, The Netherlands). With the help of the foam, the cups are isolated and thus delay the cooling. In order to minimize the cooling of the coffee in the thermos flasks as well, they are preheated before the experiments by adding boiling hot water. The water is removed from the thermos flasks shortly before the test persons arrive. By using a water dispenser (Bunn H3EA Hot Water Dispenser, Bunn, Springfield, IL, USA) for the preparation of the “very hot” coffee, constant temperatures of about 96 °C can be achieved. This coffee is prepared only after the brief introduction of the participants to keep these temperatures as long as possible.

The participants gradually mix in the test phase in an ascending fashion very hot coffee (about 96 °C) to the colder coffee (starting temperature 60 °C, 150 mL) and measure the temperature of the mixture. The added amount is standardized by a small beaker (0.04 L), which leads to a temperature increase of 2–3 °C. Then the coffee is sampled. The process of mixing is repeated by the participants until their personal optimum drinking temperature is reached, followed by the temperature, which is perceived as unpleasant, painful or too hot at which the trial is stopped. The duration of the trial is about 15–25 min.

For a pilot feasibility test of the study protocol, all students, staff and professors of the Albstadt-Sigmaringen University were invited by e-mail to participate. A total of 87 Caucasian people (65 female and 22 male; average age 33 ± 15) and thus about 5% of the university location Sigmaringen members participated in the study. The informed consent of the participants was obtained with signatures in writing following an oral information about the trial. The study protocol was approved by the ethics commission at Landesärztekammer Baden-Württemberg (Stuttgart, Germany) at 19 December 2017, Az. F-2017-094. Data was tested for normality using Shapiro–Wilk test and group comparisons were conducted using one-way analysis of variance (Minitab 16, Minitab Inc., State College, PA, USA). Statistical significance was assumed at the 0.05 significance level.

## 3. Results

The raw results of each participant are presented in Appendix B, Table A1. Descriptive statistical analysis shows that on average between participants, coffee is perceived as “too hot” from temperatures beyond 66 °C (Table 1). The standard deviation is 3 °C, which appears to be comparably low in the context of sensory analysis. The highest mentioned temperature for the pain threshold of one of the participants is 71 °C and is thus well above the specified IARC threshold of 65 °C. The range shows that the participants have a very different perception of the threshold temperature. Temperatures from 58 °C to 71 °C are determined as maximum tolerable temperatures. This results in a span of 13 °C for the pain threshold.

**Figure 2:**
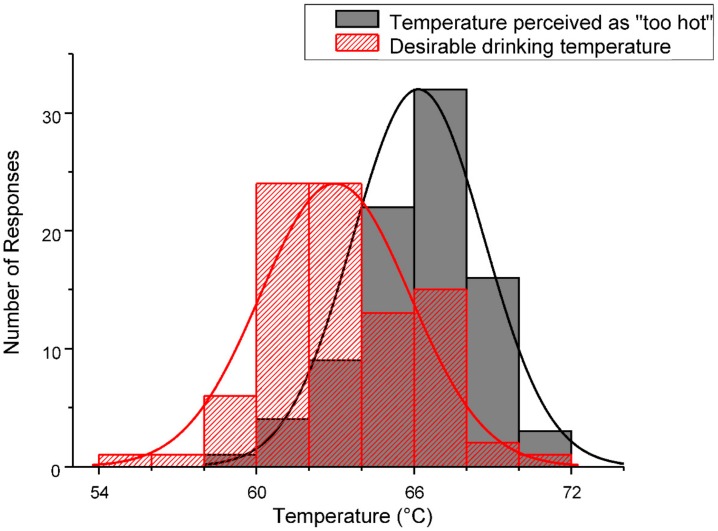
Histogram of the distributions for temperatures perceived as “too hot” and as “desirable/preferred” (*n* = 87). The curves show the normal distribution for both data sets.

Figure 2 shows the distribution of the temperatures perceived as “too hot”. Again, it can be seen that most of the participants’ responses for the pain threshold are at temperatures around 66 °C to 68 °C (*n* = 31; 36%). The category of pain threshold temperatures above 68 °C includes 19 participants (22%). Of the 87 participants, 24 persons (28%) already consider temperatures below 65 °C to be “too hot”; one participant already considered a temperature below 60 °C as being “too hot” (1%).

In addition to the maximum temperature perceived as “too hot”, the study also determined which temperatures the participants feel most desirable or preferable for coffee consumption. The descriptive statistical analysis gives a mean value of 63 °C with a standard deviation of 3 °C. The values range from a minimum of 55 °C to a maximum of 70 °C. Among the respondents are 19 participants (22%) who find their coffee at temperatures above 65 °C to be optimally temperated. Among them are two participants (2%) who even find the drinking desirable at temperatures above 68 °C. However, of all participants, a large majority of 63 persons (72%) has their personal drinking temperature below 65 °C.

The distributions for both temperatures (Figure 2) are normal. The population means are significantly different. The average difference between the desirable and pain threshold temperatures was 3 °C (standard deviation 2 °C).

## 4. Discussion

During an initial trial, the rapid cooling of the coffee and thus a difficulty of gradually increasing the temperature of the beverage was observed, especially when normal porcelain coffee cups were used. Based on previous research on cooling of coffee in different materials [26,27], the experimental setup was modified to include insulation in a foam cup holder and the use of a styrofoam cup. The cups remain in the foam holder during the experiment, only for drinking the cup is removed. Furthermore, it is important that the hot coffee for increasing the temperature is made as hot as possible, in our case using preheated thermos flasks and a hot water dispenser. Through these measures, it has been possible to almost linearly increase the temperature in the cup. Previous research has shown that the cooling behaviour of coffee is identical to the one of hot water [27], so that the study design may be transferrable to most hot beverages with similar behaviour. However, there could be an influence or interaction between acidic beverages (such as coffee) and temperature sensation, because the TRPV1 receptor integrates stimuli including heat and extracellular acidic pH [28].

Our study protocol was well usable for its purpose and no inconsistencies were observed besides one participant who already judged the initial coffee as “too hot”, but this problem can be circumvented by allowing the coffee to cool for some minutes or by decreasing the initial temperature. 

According to the literature, there may be a considerable intra-individual variation in pain threshold tests (12 °C on average [23]). While such differences were not researched in our study (only one trial per participant), the inter-individual variation in our trial is lower than this intra-individual value from the literature. This can potentially be explained by differences in the experimental design (starting from hot to cold in [23], or presenting only very few samples with large temperature differences in Refs. [24,25]). We believe that large receptor responses such as pain may hinder or restrict taste testing for some time, so that the starting point should be the low stimulus and not the high stimulus (similar to, e.g., standard procedures for taste tests for salt, acidity, etc., see ISO 8586 [29], which always start at the lowest concentration). Final evidence into this question may be only gained by testing the same beverages with the same collectives in both fashions (i.e., increasing and decreasing temperatures). This could also help to investigate the question if a hysteresis effect may occur due to the increasing temperature in the experimental design.

The strength of coffee may also play a role in the sensation of temperature [23]. However, only one single coffee type was used in this study and the coffee in the study by Lee and O’Mahony [23] was much stronger. Coffee type may therefore potentially explain the larger variances in Lee and O’Mahony [23] compared to this study. A larger bitterness response (or pH decrease, see above), for example, may interact with the temperature response. Interactions may occur between irritant stimuli (heat or cold) and the human taste system either at the peripheral level (receptor level which is unlikely), or the central nervous system level.

In addition, this study does not assess whether there are differences between consumers drinking their coffee with milk/cream/sugar or black without additions. Therefore, the protocol could be expanded in the future by including tastants (such as sourness and bitterness in the coffee) and flavourants. While the perceived intensity of basic tastes does not strictly increase with temperature, this is true for some flavours. Caffeine is also a bioactive compound, which may lead to individual reactions due to genetics (the influence of caffeine could be tested by comparison with decaffeinated coffee). Before further use, the protocol should be validated as to whether reproducible results are achieved both intra-individual and inter-individual within taste-testing panels and for different beverage types and preparations. A potential limitation of the protocol may also be the three-category scale for descriptors, which could possibly be expanded using a larger number of categories or a numeric pain rating scale.

The results of the pilot test study show that, on average, the participants judge coffee as being “too hot” just above the threshold of 65 °C suggested by IARC for “very hot” beverages that are probably carcinogenic to humans [1,2]. This consumption preference of German consumers may explain the fact that an increased incidence of oesophageal cancer has not been described in connection to hot beverage consumption in Germany, while in other countries, such as for tea in Iran or mate in South America, where such epidemiological associations were mainly described, the preferred consumption temperature was typically much higher than 70 °C [3,4,5,6,7,8]. All other previous studies were conducted in the USA, and very similar consumer preference was detected in this country as in our study (ideal consumption temperature 63–68 °C [24]; optimal drinking temperature 58 °C [19]; most preferred serving temperature 75 °C [25]; mean preferred temperature for drinking 60 °C [23]; preferred temperature of ingestion 56 °C [22]).

Nevertheless, some participants in Germany tolerate temperatures well above 70 °C and thus may be exposed to an increased risk of oesophageal cancer. Therefore, the question arises how this group of consumers can tolerate such high temperatures without experiencing pain.

According to Lee et al. [18], the threshold for sensation of pain on the tongue starts at temperatures of about 46–48 °C, while the TRPV1 channels open at 43 °C. However, the average coffee drinking temperatures in our study clearly exceed this level. Obviously, the liquid has much higher temperatures than the surface of the tongue when heated by the coffee. But measurements of Lee et al. [18] showed that coffee at a temperature of 60 °C may raise the surface of the tongue to 53 °C, which is above the postulated pain threshold of the tongue.

Still, many of the participants in our study perceive coffee temperatures of around 60 °C and also much higher as desirable and certainly not too hot. This discrepancy is explained by Lee et al. [18] by the fact that the temperatures may not be kept in the mouth long enough. A swallowing process lasts only a few seconds, so the temperature exposure acting on the mucous membranes in the mouth and on the tongue is not sufficiently long and thus not able to reach an action potential. Thus, no pain is transmitted despite high temperatures, so that consumers are able to drink the hot drinks without pain.

Finally, another hypothesis is that the pain potential can possibly be reduced by a habituation effect through continuous hot beverage consumption over the lifetime [30]. Lütgendorff-Gyllenstorm [31] assumes that the defence mechanism or instinct against consumption of very hot foods and beverages, still present in infancy, is lost during growing up to adulthood by education (as being the desirable consumption behaviour) and gradual adaptation to increased temperatures. Hence, this habituation effect may also explain the large inter-individual differences of up to 15 °C.

Some previous studies considered the question whether temperatures of very hot beverages actually influence cancer risk or other disease outcomes [1,3,21,22]. In fact, previous experimental studies have shown that some consumers may ingest their coffee at temperatures that can burn the epithelium of the oesophagus [23]. It has been shown that the participants on average perceive coffee most preferable at temperatures of about 60 °C. People who drink their coffee black prefer slightly higher temperatures than participants who drink their coffee with milk. Similarly, “weaker” (less concentrated) coffee is preferred at higher temperatures [23]. Our findings basically corroborate these observations, suggesting that the temperature at which coffee is considered preferable in Germany is typically <65 °C, and hence below the threshold for carcinogenic risk [1,2].

## 5. Conclusions

The developed method is well-suitable for the purpose to obtain temperature preferences of beverages and next steps could include intra- and inter-individual validation work and field testing with larger collectives of participants. After final validation, the method could be useful for testing temperature threshold differences in oesophageal cancer patients versus a control group.

## Figures and Tables

**Table 1:** Summarized results of the pilot study (*n* = 87).

Temperature	Mean	Standard Deviation	Minimum	Maximum	Range	Median
Pain threshold ^a^	66 °C	3 °C	58 °C	71 °C	13 °C	66 °C
Preferred drinking temperature	63 °C	3 °C	55 °C	70 °C	15 °C	63 °C

^a^ Temperature perceived as “too hot”.

## Appendix B

**Table A1:** Raw results of pilot study (*n* = 87).

Gender	Age	Temperature Perceived as “Too Hot” (°C)	Preferred Drinking Temperature (°C)	Coffee Consumption Behaviour (Cups per Day)
male	55	58.0	55.0	2–3
female	45	60.2	<60.0	2–3
male	56	60.2	57.4	>4
female	28	61.5	59.7	2–3
male	35	61.9	58.4	>4
male	24	62.0	60.0	2–3
male	49	62.0	60.0	2–3
female	22	62.4	59.0	1
female	21	63.0	59.6	2–3
male	28	63.0	60.5	2–3
female	22	63.2	61.2	>4
female	19	63.3	61.7	seldom
female	27	63.5	60.8	2–3
female	23	63.8	61.0	2–3
female	34	64.0	61.0	2–3
female	21	64.1	62.2	2–3
female	39	64.1	60.4	2–3
female	23	64.2	62.4	1
male	25	64.2	62.0	2–3
male	63	64.3	62.3	2–3
female	21	64.5	61.7	seldom
male	55	64.5	60.0	>4
female	34	64.8	62.6	2–3
female	38	64.9	58.0	>4
female	24	65.0	63.0	2–3
male	21	65.0	62.5	2–3
female	20	65.2	63.6	seldom
male	72	65.2	61.7	>4
male	22	65.3	63.3	2–3
female	21	65.4	62.5	2–3
female	22	65.4	61.9	>4
female	22	65.6	61.0	2–3
female	23	65.7	62.0	2–3
female	52	65.7	60.2	>4
female	26	65.8	63.0	>4
female	53	65.9	59.3	seldom
female	59	66.0	60.0	>4
male	22	66.0	61.5	2–3
female	26	66.1	60.0	seldom
female	36	66.2	64.0	2–3
female	22	66.3	63.5	2–3
male	53	66.3	60.0	>4
female	22	66.4	61.0	>4
female	23	66.4	64.5	1
female	24	66.5	63.0	2–3
female	41	66.7	60.5	2–3
female	25	66.9	63.0	>4
female	23	67.0	62.0	1
female	19	67.1	65.0	2–3
female	20	67.1	63.4	2–3
female	51	67.1	64.0	2–3
male	52	67.1	61.0	>4
female	21	67.3	65.0	>4
female	23	67.3	66.0	2–3
female	25	67.3	64.5	>4
female	51	67.3	66.0	>4
male	35	67.3	65.0	>4
male	42	67.3	65.0	2–3
female	25	67.4	66.0	1
male	42	67.4	60.0	>4
female	24	67.5	67.3	>4
female	19	67.6	65.0	seldom
female	24	67.6	66.0	2–3
female	48	67.7	63.3	2–3
female	55	67.8	63.7	>4
male	22	67.8	63.4	2–3
female	54	67.9	67.4	>4
female	62	67.9	63.8	2–3
female	58	68.0	63.3	2–3
female	22	68.2	64.6	2–3
female	21	68.3	64.5	2–3
male	64	68.4	62.8	>4
male	20	68.5	66.0	2–3
female	51	68.6	67.5	2–3
female	22	68.8	68.2	>4
female	56	68.9	67.0	2–3
female	21	69.0	66.0	2–3
female	24	69.0	63.2	2–3
female	24	69.0	64.0	seldom
female	48	69.3	67.0	>4
female	19	69.5	66.1	2–3
female	23	69.5	66.0	2–3
female	29	69.6	67.7	>4
female	22	69.8	67.6	2–3
female	55	70.2	69.3	2–3
male	30	70.5	65.6	>4
female	23	71.2	70.0	>4

Data sorted according to temperature perceived as “too hot”.
